# Phasic Firing in Vasopressin Cells: Understanding Its Functional Significance through Computational Models

**DOI:** 10.1371/journal.pcbi.1002740

**Published:** 2012-10-18

**Authors:** Duncan J. MacGregor, Gareth Leng

**Affiliations:** Centre for Integrative Physiology, University of Edinburgh, Edinburgh, United Kingdom; University of Oxford, United Kingdom

## Abstract

Vasopressin neurons, responding to input generated by osmotic pressure, use an intrinsic mechanism to shift from slow irregular firing to a distinct phasic pattern, consisting of long bursts and silences lasting tens of seconds. With increased input, bursts lengthen, eventually shifting to continuous firing. The phasic activity remains asynchronous across the cells and is not reflected in the population output signal. Here we have used a computational vasopressin neuron model to investigate the functional significance of the phasic firing pattern. We generated a concise model of the synaptic input driven spike firing mechanism that gives a close quantitative match to vasopressin neuron spike activity recorded *in vivo*, tested against endogenous activity and experimental interventions. The integrate-and-fire based model provides a simple physiological explanation of the phasic firing mechanism involving an activity-dependent slow depolarising afterpotential (DAP) generated by a calcium-inactivated potassium leak current. This is modulated by the slower, opposing, action of activity-dependent dendritic dynorphin release, which inactivates the DAP, the opposing effects generating successive periods of bursting and silence. Model cells are not spontaneously active, but fire when perturbed by random perturbations mimicking synaptic input. We constructed one population of such phasic neurons, and another population of similar cells but which lacked the ability to fire phasically. We then studied how these two populations differed in the way that they encoded changes in afferent inputs. By comparison with the non-phasic population, the phasic population responds linearly to increases in tonic synaptic input. Non-phasic cells respond to transient elevations in synaptic input in a way that strongly depends on background activity levels, phasic cells in a way that is independent of background levels, and show a similar strong linearization of the response. These findings show large differences in information coding between the populations, and apparent functional advantages of asynchronous phasic firing.

## Author Summary

Vasopressin is a hormone secreted from specialised brain cells into the bloodstream, acting at the kidneys to control water excretion, and thereby help regulate osmotic pressure. This is a cell membrane property determined by the ratio between body salt and water, and its maintenance is essential to the function of all our cells and organs, which depend on a stable fluid volume and extracellular salt concentration. Specialised cells in the brain sense osmotic pressure and generate electrical signals, which the thousands of vasopressin neurons process and respond to by producing and secreting vasopressin. The individual vasopressin cells generate an interesting phasic pattern of electrical activity in response to rises in osmotic pressure – they fire in long bursts, separated by long silences. In our project we're using modelling to simulate this phasic pattern of electrical activity and how it relates to the input signals, trying to understand exactly why vasopressin cells generate this kind of pattern and exactly what advantages it offers to signal processing and the control of vasopressin secretion.

## Introduction

Magnocellular vasopressin neurons produce and secrete the antidiuretic hormone vasopressin in response to increases in the osmotic pressure of extracellular fluid [1]. They form a key part of the highly robust homeostatic system which maintains osmotic pressure within narrow bounds. Each of the neurons independently encodes and responds to an input signal, but they must also coordinate as a population, making the vasopressin neurons a prime example of a distributed control system [2].

The vasopressin cell bodies, located in the supraoptic and paraventricular nuclei of the hypothalamus, project axons to the posterior pituitary gland, and receive synaptic input from osmosensitive neurons located near the third ventricle, as well as responding through depolarising currents generated by the vasopressin neurons' own osmosensitive ion channels [2]–[4]. The generated action potentials (spikes) propagate down the axons to trigger hormone secretion from the axonal terminals into the blood. When osmotic pressure rises, the secreted vasopressin acts at the kidneys to reduce the amount of water lost in urine.

After chronic water deprivation, many vasopressin neurons respond to the increased osmotic input by shifting from slow irregular firing into a distinctive phasic pattern, consisting of long bursts and silences lasting tens of seconds. As input increases the bursts lengthen, eventually shifting to fast continuous firing [3], [4]. Unlike the rhythmic activity observed in many other neural systems, phasic firing is generated by an intrinsic mechanism, rather than by network activity [5]. The vasopressin neurons have no synaptic interconnections, and they fire asynchronously; accordingly, the hormone output reflects a smooth population signal rather than the fluctuating activity at individual cells. Activity-dependent secretion is characterised by a combination of *frequency facilitation*, whereby disproportionately more vasopressin is secreted at higher frequencies of stimulation, and *fatigue*, whereby peak secretion rates can only be sustained transiently. As a result, phasic firing patterns are optimally efficient [6], [7], in the sense of maintaining a given level of secretion with the fewest spikes.

However, phasic firing is efficient only because the properties of the neurosecretory terminals make it so, and those properties are not universal – in particular the properties of oxytocin terminals in the posterior pituitary gland are different from those of vasopressin terminals, and seem to be adapted to the different firing properties of oxytocin neurons. Both cells' secretion mechanisms are subject to facilitation, and oxytocin cells can also show an enhanced response to phasic firing compared to a continuous pattern [8]. However, the phasic pattern is not optimal as it is for vasopressin cells. The oxytocin cells have a much greater facilitation frequency range, and are not subject to short timescale (10s of seconds) fatigue [9].

It seems that the secretion mechanism is as much adapted to the spike patterning as vice versa, thus while phasic firing is efficient for secreting vasopressin, we must look deeper to fully understand why these neurons fire phasically. To address this, we need a model that can accurately reproduce the range of firing response observed *in vivo*, which we can then systematically interrogate to understand how phasic firing affects the signal processing properties of the neurons. This requires a model which includes the essential elements of the neuronal firing mechanism, but which is still simple enough to manipulate and be well understood.

The core of the phasic firing mechanism is a slow depolarising after potential (DAP), acting on a timescale of several seconds. This activity-dependent current increases neuronal excitability, generating a positive feedback effect. When a vasopressin cell is in a silent phase, a few random close spikes will generate the beginnings of a depolarised ‘plateau potential’ that can self-sustain, resulting in a prolonged burst of spikes. However, during this burst, firing also triggers the dendritic release of the opioid peptide dynorphin [10], which acts back on the cell of origin in an autocrine manner to progressively attenuate the DAP [11]. The cumulative effect of activity-dependent dynorphin secretion eventually causes the plateau to fail, and the cell begins a new silent phase [5].

There are two current theories for the slow DAP. Li and Hatton [12] suggest that it is caused by the removal of a hyperpolarising K^+^ leak current, whereas Bourque *et al* [13], [14] suggest that a depolarising non-specific cation current is responsible. Using a Hodgkin-Huxley based model fitted to *in vitro* data, Roper *et al* [15], [16] argue that the generation of both a plateau, and a silent period, is more easily explained by the K^+^ leak based mechanism. The Roper model uses only one compartment, but includes a dynorphin mechanism, and was the first published model to demonstrate bursting. However, the Roper model is based on data recorded *in vitro*. Vasopressin cells recorded *in vitro* are largely denuded of afferent input, and accordingly have a high input resistance; this directly impacts upon membrane time constants, and all activity-dependent potentials are amplified [17]. In particular, the DAP following single spikes *in vitro* is so large that it can produce regenerative spiking, while perturbations produced by synaptic input are relatively sparse. For vasopressin cells *in vitro*, bursts comprise spikes that occur at a relatively constant inter-spike interval, giving a symmetrical distribution of intervals with a modal value that is the inverse of the mean intraburst firing rate. This distribution implies regenerative spiking. By contrast, *in vivo*, bursts in vasopressin cells comprise inter-spike intervals that have a very skewed distribution, with a mode that is disproportionately short for the mean firing rate – and which is largely independent of the mean firing rate. At the same time, the distributions have a very long tail, and this tail can be fit by a single negative exponential. From these features it can be deduced that, *in vivo*, neuronal excitability after a spike follows a sequence of hypoexcitability – consistent with an HAP, followed by hyperexcitability, consistent with a subthreshold DAP peaking at ∼40 ms. However, most spikes occur at longer intervals, and the negative exponential distribution suggests that their arrival is the result of a Poisson process – and is random in being independent of prior activity. Thus *in vivo*, spiking is not regenerative, and spike patterning is dominated by the stochastic effects of synaptic input [18]; these effects not only increase variation and noise in the output but can also change the qualitative behaviour [19]. In phasic cells in particular, small variations in firing activity can trigger the starting and stopping of bursts [20], [21].

Nadeau *et al.* [22] extended the Roper model by adding synaptic input and a simulation of direct osmosensitivity. In this model, synaptic noise produces some variability in spike timing, but the burst mechanism remains essentially regenerative: bursts, once triggered, can be sustained in the absence of synaptic input, and firing within bursts has the extreme regularity typical of *in vitro* data but very different to *in vivo* data. Thus intraburst frequency is largely unaffected by EPSP or IPSP rates, except at high frequencies of synaptic input, which give reasonable matches to ISI distributions observed *in vivo* but produce continuous, rather than phasic, firing.

Clayton *et al.* [23] took a different approach; they use an integrate-and-fire model and ask what is the simplest model that can fit *in vivo* spike data to a point whereby data from a model cell cannot be distinguished statistically from data from a target vasopressin cell? In this model, the combination of a slow DAP and the opposing action of dynorphin is represented by an explicit bistable mechanism which drives phasic firing. Using automated parameter fitting, this model produces extremely close fits to *in vivo* spike patterns, and can be fitted well to cells firing phasically, or firing continuously. However, we observed that, when a model cell with parameters that fit a phasically firing cell is challenged with increasing input, it fails to shift to continuous firing. Thus the Clayton model's explicit bistable mechanism captures the neuron's behaviour concisely, but within only a limited range. This suggests that some of the fitted parameters, particularly those accounting for bistability, are activity-or input dependent, and rather than being parameters, need to be incorporated into the model's dynamics.

Here we simulate vasopressin neurons in a model that displays emergent bistable behaviour, combining the best elements of previous models. The model gives a more complete match to vasopressin neuronal firing activity, while being simpler and more directly related to the physiology. We then use this model to explore how vasopressin cell activity encodes afferent signals, by comparing a population of phasically firing model neurons with an otherwise identical non-phasic population. We show that bistability and phasic firing gives neurons acting as a population several important signal processing properties that non-phasic neurons lack. They can produce a strongly linear response to both a constant and transient input signal, and they produce a consistent response to transient signals, independent of background activity. These are important properties that have been identified in the vasopressin response *in vivo* [1], and may also apply more generally to neural signal processing.

## Methods

### The model

The model takes as its base the oxytocin cell model of [19], [24]. This is a leaky integrate-and-fire (IGF) model driven by Poisson random decaying input perturbations simulating synaptic input. A fast large hyperpolarising afterpotential (HAP) and a slow small after-hyperpolarising potential (AHP), are simulated as exponentially decaying variables, incremented (using a negative value for hyperpolarisation) when a spike is fired. These are summed with a fixed resting potential and the synaptic input to give the membrane voltage. When this crosses the spike threshold a spike is recorded, followed by an absolute refractory period of 3 ms. This gives a close match to the *in vivo* spike patterning in oxytocin neurons, and by adding a simple fast DAP, using the same decaying exponential form, a similar model can closely match the intraburst activity of vasopressin neurons.

These representations of post-spike potentials were developed to match the spike-dependent changes in excitability deduced from the interspike interval (ISI) distributions and hazard functions of oxytocin and vasopressin cells recorded *in vivo*, based on the decaying post-spike depolarisation and hyperpolarisations observed in *in vitro* intracellular recordings. They are comparable to the forms used in Roper's Hodgkin-Huxley based model [15], [16], which represents the HAP, AHP, and DAP as separate compartments of intracellular [Ca^2+^], ([Ca^2+^]_i_) driving Ca^2+^ sensitive currents. The varied decay time courses used in the IGF model are similar to the corresponding compartmental [Ca^2+^] half-lives.

**Figure 1:**
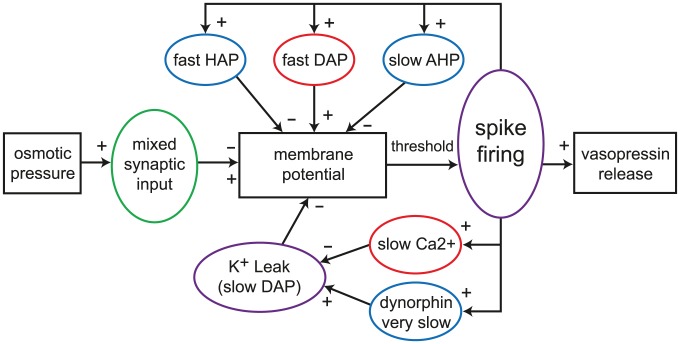
The vasopressin spike firing model. Schematic illustrating the structure of the integrate-and-fire based single neuron spiking model. Input is a Poisson random timed mix of excitatory and inhibitory pulses, simulating PSPs. These are summed to generate a membrane potential which is also modulated by a set of spike triggered Ca^2+^ based potentials. The HAP, fast DAP and AHP are based on simple decaying exponentials, similar to a previous oxytocin cell model [19], [24]. The K^+^ leak current based slow DAP which generates bursting is based on the mechanism of the Hodgkin-Huxley type model of [16]. Spikes are generated when the membrane potential crosses a threshold value.

We explored whether adding a second, slower, simple DAP could generate quantitatively realistic burst firing in the IGF model. A sustained plateau could be achieved if the DAP half life was >2 s, and combined with saturation to limit the DAP magnitude. Given the ability to sustain a plateau, an activity-dependent mechanism is required to terminate the bursts. Physiologically, this involves spike-dependent release of dynorphin which inhibits the DAP. Using a slow spike-dependent exponentially decaying variable to inhibit the DAP, combined with a hyperpolarised resting potential (−75 mV), we could produce bursts, but could not achieve sharp bistable switches in activity, and could not produce *in vivo* comparable silent periods, only periods of slower activity. The Roper model [15], [16] uses a different DAP mechanism to solve these problems; the burst plateau is generated by fully suppressing a hyperpolarising K^+^ leak current that is partially active at resting potential, and silences are periods where the K^+^ leak current is fully active, suppressing firing. This single mechanism can generate both activity dependent depolarisation and hyperpolarisation. Its model form, fitted to *in vitro* data, includes saturation and a simple relation between competing spike-triggered increases in [Ca^2+^]_i_ and dynorphin, allowing dynorphin accumulation to eventually switch off a burst and generate a prolonged silence. This mechanism was simplified and integrated into the IGF model to produce the design illustrated in Figure 1.

### Model equations

Twin Poisson random processes generate excitatory and inhibitory post-synaptic potential (EPSP and IPSP) counts *e_n_* and *i_n_* at each 1-ms time step, using mean rates *I*_re_ and *I*_ri_; the IPSP frequency *I*_ri_ is defined as a proportion of *I*_re_ given by *I*_ratio_. All of the results here use *I*_ratio_ = 1 so that input rate is controlled by using just *I*_re_. The input potentials have fixed amplitudes *e*_h_ = 2 mV and *i*_h_ = −2 mV and are summed to give the input:
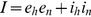
(1)This is summed with the synaptic component of the membrane potential, *V_syn_*, decaying exponentially with half life λ*_syn_*:
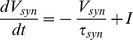
(2)Time constants are calculated from half-life parameters by:
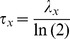
where *x* is the variable concerned.

Variables for the HAP, AHP and the fast DAP decay exponentially, controlled by half-life parameters, λ*_HAP_*, λ*_AHP_*, and λ*_DAP_*, and are incremented by *k_HAP_*, *k_AHP_*, and *k_DAP_* when a spike is fired. The AHP also depends on [Ca^2+^]_i_, so that only fast spiking substantially activates the AHP.
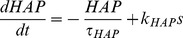
(3)

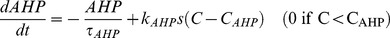
(4)

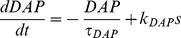
(5)where *s* = 1 if a spike is fired at time *t*, and *s* = 0 otherwise.

Variables for [Ca^2+^]_i_, *C* and dynorphin, *D*, use similar forms:
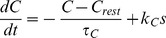
(6)

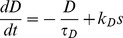
(7)The slow DAP (*L*) uses a simplified version of the DAP conductance equations in the Roper model [14], [15], defined as two components:
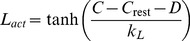
(8)where *k_L_* is a scaling parameter. The function tanh, previously used in the fit to *in vitro* data [15], ensures that the activation of *L* is sigmoidal, with half maximal activation at 0. This is used to inhibit the K^+^ leak potential, *V_L_*, scaled by conductance parameter *g_L_*:
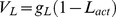
(9)Finally, these components are summed with the resting potential, *V_rest_*, to give the membrane potential *V*:
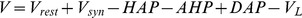
(10)When *V* exceeds the spike threshold, *V_thresh_*, a spike is fired, though its form is not modelled.

The parameter values for the figures in this paper are given in tables 1 and 2.

**Table 1:** Single neuron model parameters fitted to cell v1 (a.u., arbitrary units).

Name	Description	Value	Units
*I*_re_	excitatory input rate	600	Hz
*I*_ratio_	inhibitory input ratio	1	-
*e*_h_	EPSP amplitude	2	mV
*i*_h_	IPSP amplitude	−2	mV
λ*_syn_*	PSP half life	7.5	ms
*k_HAP_*	HAP amplitude per spike	60	mV
λ*_HAP_*	HAP half life	8	ms
*k_DAP_*	fast DAP amplitude per spike	0	mV
λ*_DAP_*	fast DAP half life	150	ms
*k_AHP_*	AHP activation factor	0.00012	mV/nM
λ*_AHP_*	AHP half life	10000	ms
*C_AHP_*	minimum [Ca]_i_ to activate AHP	200	nM
*C*_rest_	rest [Ca]_i_	113	nM
*k_C_*	[Ca]_i_ increase per spike	10	nM
λ*_C_*	[Ca]_i_ half life	2500	ms
*k_D_*	dynorphin activation per spike	1.68	a.u.
λ*_D_*	dynorphin half life	10000	ms
*k_L_*	K^+^ leak calcium sensitivity	36	nM
*g_L_*	K^+^ leak maximum voltage	8.5	mV
*V*_rest_	resting potential	−56	mV
*V*_thresh_	spike threshold potential	−50	mV

**Table 2:** Model parameters fitted to *in vivo* cells.

Cell	Model	*I*_re_	λ*_HAP_*	*k_DAP_*	*k_AHP_*	*k_C_*	*k_D_*	λ*_D_*	*g_L_*
v1	m1	600	8.0	0.00	0.00012	10.0	1.68	10000	8.5
v2	m2	1050	10.5	1.15	0.00017	11.8	2.79	7500	8.0
v3	m3	920	9.5	1.20	0.00005	12.0	3.10	7500	8.0
v4	m4	630	10.5	1.00	0.00013	12.0	1.95	10000	10.5
v5	m5	530	8.5	0.90	0.00004	12.0	2.15	10000	8.5

We also tested a more complex model of dynorphin dynamics where release depends on the availability of dynorphin for activity-dependent release, which in turn depends on a slow activity-dependent mechanism, *T*, possibly representing vesicle translocation from some reserve pool to the releasable pool close to the membrane, similar to the mechanism suggested for vesicles at the terminal release sites [25]:
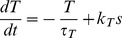
(11)The releasable store (*D*_store_) accumulates at a rate determined by *T*, and is capped at parameter *D*_storecap_ (D_store_ increases only if D_store_<D_storecap_). Dynorphin release (and store depletion) per spike is independent of the store, unless *D*_store_ is too low for one unit of release, defined by parameter *D*_spike_:
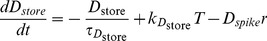
(12)

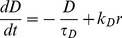
(13)where *r* = 1 if *s* = 1 and *D*_store_>*D*_spike_, and *r* = 0 otherwise. This is incorporated into the model by replacing eqn.7 with eqn.13.

### Simulating osmotic input

To simulate the effect of an acute systemic intraperitoneal injection of hypertonic saline, which has a delayed effect on osmotic pressure as the hypertonic saline enters the blood [26], we added an osmotic pressure variable *O* which shifts towards parameter *O*_inject_ with time constant τ*_O_*:
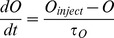
(14)where initially *O*_inject_ = *O*. Injection is simulated by changing *O*_inject_ at a specified time. Synaptic input is then generated assuming a simple linear relation:
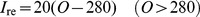
(15)which with *I*_ratio_ = 1, gives a parallel increase in excitatory and inhibitory synaptic input with increased osmotic pressure. The values 20 and 280 scale *I*_re_ so that the physiological 1–8spikes/s range corresponds to osmotic pressures of ∼290–320 mOsmol/l.

### Spike pattern analysis

The data from the model and from experimental recordings consist of series of ISIs. These are used to calculate mean firing rates and to generate ISI histograms and hazard plots, as described in [18]. A burst detection algorithm is used to detect and measure bursts, with a burst defined as a train of >25 spikes with no interval >1500 ms. Burst measures include spike count, burst duration, silence duration, and intra-burst firing rate. The *in vivo* spike data for model fitting are from extracellular recordings of magnocellular vasopressin neurons, recorded from the supraoptic nucleus in urethane-anaesthetised rats. The method is detailed in Sabatier *et al.* [18].

### Pulse response

To test the model neuron response to transient changes in input we used a fixed synaptic input rate (*I*_re_) as a background and added four identical 1-s duration changes to this rate at intervals of 100 s. We measured the response as the mean firing rate over the four 1-s pulses.

### Simulating cell heterogeneity

To simulate *in vivo* data gathered from multiple cells with varied spiking patterns, we took a set of parameters based on an existing fit of the model, and added random variation to the parameter subset which we vary to fit recorded cells (see Results below). Each varied parameter was defined by a fixed mean and standard deviation, using these to generate normally distributed random values.

## Results

### Analysis of *in vivo* phasic firing activity

**Figure 2:**
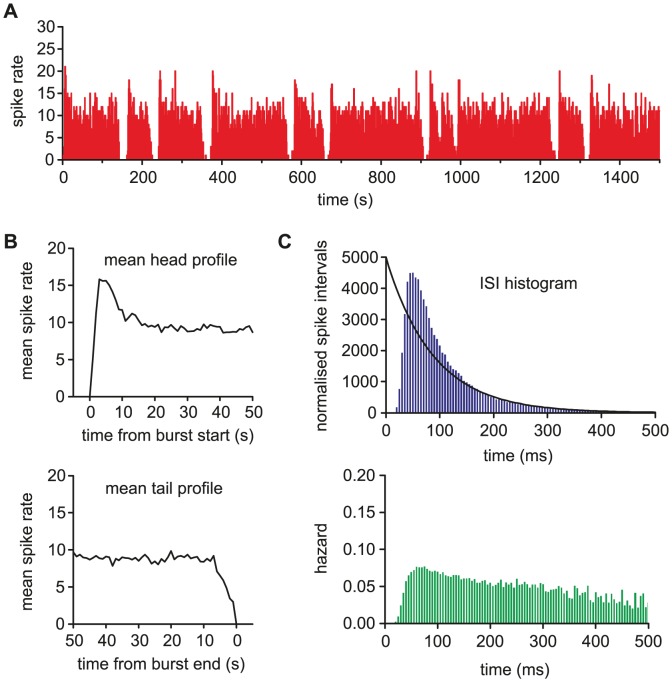
Analysis of *in vivo* phasic firing activity. (**A**) The 1-s bin spike rate counts for a phasic firing vasopressin neuron recorded in rat supraoptic nucleus. The cell fires in long bursts of varying duration (here mean 154s) which begin with a distinct peak before falling to a more stable intraburst firing rate (mean 8.91Hz). Bursts are separated by silent periods of almost no firing, and more regular duration (here mean 16s). (**B**) The burst profile of a cell is characterised by examining the mean firing rate over the first 50s and last 50s across all bursts, defining a mean shape for the head and tail. At the start of a burst, the firing rate rises rapidly, over ∼3s before falling back to a relatively stable plateau. The burst tail shows a slightly less rapid shift, declining over ∼7s. (**C**) The ISI histogram and hazard function show the short term spike patterning and post-spike excitability. The lack of short intervals is due to the HAP dominated refractory period. The following peak in excitability indicates a DAP. Beyond ∼150ms, the tail of the histogram can be fitted by a decaying exponential (y = 5000e^−0.0114x^, r^2^ = 0.982), indicating that firing is otherwise random. The single spike effect of the AHP is too small to distinguish here [24].

To match the firing activity of a cell with a model in a quantitatively robust way, we need to define the features of that activity in a way that we can statistically compare recorded and model generated data. Spike activity comprises the overall firing pattern, shown by spike rate as measured in bins of varying width; the bursting characteristics, using burst detection to quantify bursts and silences and plot the mean burst profile; and short term spike patterning and excitability, contained in the ISI distribution and related hazard function (Figure 2). Together, these measures capture the important features of phasic firing, and describe the variation between individual cells. They also relate closely to many of the underlying mechanisms, such as the post-spike potentials.

These burst measures differ considerably between cells, and in any one cell, as synaptic input increases, bursts lengthen, silences shorten and the intra-burst firing rate increases [3], [4]. The burst profile of a cell typically shows a distinctive peak in firing rate at the start of the burst which rapidly rises and then declines to a plateau (Figure 2B). *In vitro* experiments have shown that this decline requires an AHP [27].

The ISI histogram and hazard plots (Figure 2C), used to examine spike patterning within bursts, show a relative refractory period of 30–50 ms followed by a period of increased firing excitability, which slowly decays, consistent with the superposition of an HAP and a slower DAP. The long exponentially decaying tail of the histogram suggests that after about 150 ms has elapsed since a spike, spikes arise randomly, indicating that there is no patterning in the synaptic input activity.

### Fitting the model to *in vivo* burst firing activity

Our previous *in vivo* modelling work [23] used automated parameter fitting based on a genetic algorithm. The present model can be fitted with the same technique (not shown), but is also simple enough to fit manually. Using manual fitting helps to understand how each parameter effects spiking behaviour, and also how parameters interact and how independent they are.

To fit the model to data, we began with the ISI distribution and hazard function, and then moved to the burst features. The basic membrane parameters, for *V_rest_*, *V_thresh_*, PSP magnitude, and PSP half life, are derived from the earlier oxytocin cell model [19]; oxytocin cells are closely related to vasopressin cells but lack a DAP and a bistable burst mechanism. The fitting for each *in vivo* recording began with *g_L_* = 0, to switch off bursting and get an initial approximate fit for the synaptic input rate, HAP, AHP and fast DAP parameters; the cell's intra-burst ISI distribution, hazard function and firing rate can be closely fit without producing bursting. The burst mechanism was then turned on by setting *g_L_*, and the full model fitted to the mean firing rate, mean burst duration, mean silence duration, and mean burst profile.

**Figure 3:**
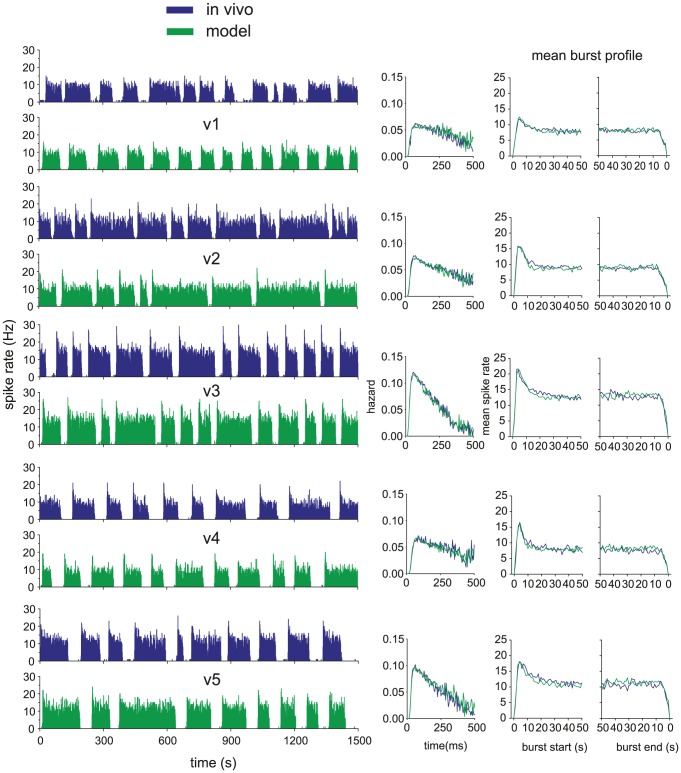
The model fitted to five typical phasic cells recorded *in vivo*. On the left we show pairs of matched *in vivo* and model generated spike rate data, and on the right, the fitted hazard and burst profiles. The model closely matches burst profile, mean burst length, mean silence length, intraburst firing rate, and the intraburst hazard, showing post-spike excitability and patterning. A subset of eight of the model's 21 parameters were varied to match the cells. The fit parameters vary synaptic input rate, HAP half life, AHP magnitude, fast DAP magnitude, dynorphin magnitude, calcium magnitude and K+ leak conductance. The parameter values are given in tables 1 and 2.

To understand which parameters are most important to variation between cells, we attempted to fit different cells by varying as few parameters as possible. This process identified the dynorphin parameters *k_D_*, λ*_D_*, calcium parameter *k_C_*, and *g_L_* as those required to fit the burst measures. Parameter *k_D_* dominates the burst duration, while λ*_D_* and *g_L_* can be adjusted to match varied silence durations. The HAP, AHP and DAP parameters were further adjusted to compensate for the K^+^ leak current's additional effect on short term spike patterning. The AHP parameter *k_AHP_* dominates the size of the peak in firing rate at the start of each burst, consistent with experimental evidence that this current is responsible [27]. Five representative fits to recorded phasic cells are shown in Figure 3, using the parameters given in Tables 1 and 2; all give a close match to the firing rate, hazard, and burst measures (Table 3).

**Table 3:** Fitted model (m) and *in vivo* (v) burst measures.

Data	Intra(Hz)	Burst Mean(s)	Burst SD	Silence Mean(s)	Silence SD
v1	7.89	85	31	41	23
m1	7.90	85	51	38	5
v2	8.91	154	68	16	4
m2	8.88	149	93	19	3
v3	12.85	88	37	29	5
m3	12.87	83	51	26	3
v4	8.05	112	39	50	10
m4	8.03	107	54	47	8
v5	11.00	100	38	51	22
m5	11.06	92	55	49	6

### Examining the model's burst firing mechanism

**Figure 4:**
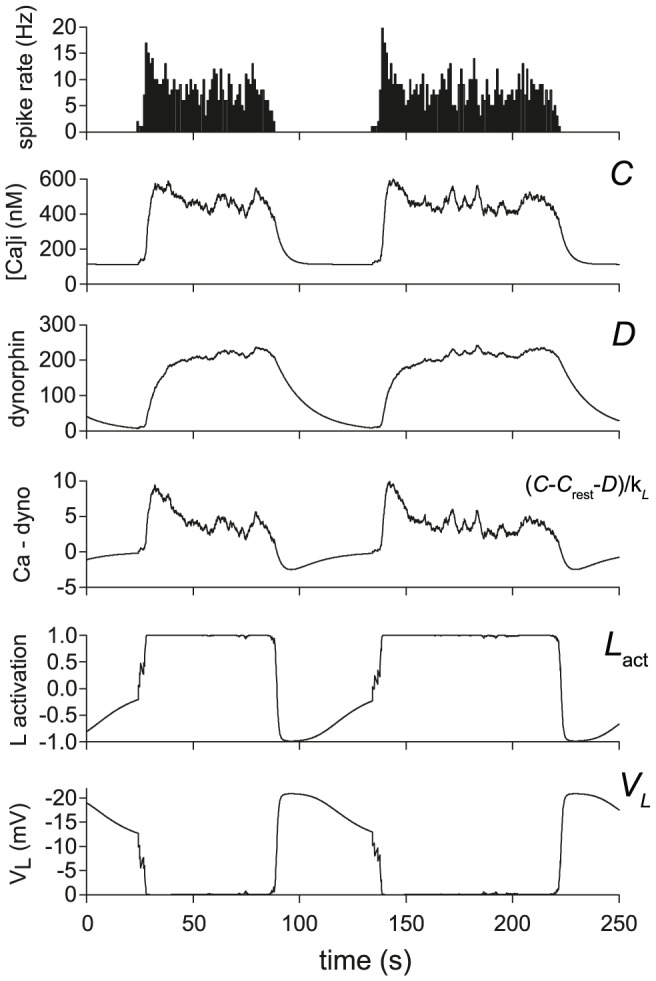
The model's burst firing mechanism. The data here shows two typical bursts from the model fitted to cell v4. The burst mechanism is driven by the spike triggered accumulation of [Ca^2+^]_i_ and dynorphin. The [Ca^2+^]_i_ signal inhibits the hyperpolarising K^+^ leak current, increasing firing and creating a positive feedback that sustains a burst. The more slowly accumulating dynorphin signal opposes the effect of [Ca^2+^]_i_, eventually causing burst termination and driving a silent period of sustained hyperpolarisation. The positive feedback combined with the two opposing effects acting on different timescales creates an emergent bistability, shown in the rapid shifts of the K^+^ leak (*L*) activation and the resulting effect on membrane potential (*V_L_*).

The bistable burst firing mechanism is based on opposing effects of [Ca^2+^]_i_ and dynorphin, acting on different timescales. These do not act deterministically, but shift the probability of a burst starting or stopping, subject to the stochastic synaptic input. When rapid successive spikes arise during a silent period, they cause an increase in [Ca^2+^]_i_ which begins to suppress the hyperpolarising K^+^ leak current. This triggers activity-dependent positive feedback, increasing firing rate and hence [Ca^2+^]_i_. This feedback becomes self-sustaining, maintaining the suppression of the leak current and hence allowing spike activity to be sustained at a relatively stable level (Figure 4).

As a burst begins, the AHP begins to accumulate, but this rises more slowly than the burst initiates, allowing a peak in firing rate at the head of the burst, before the AHP slows the intra-burst firing. Bursts reach a stable state when activity-dependent inhibition reaches an approximate equilibrium with activity-dependent disinhibition of the leak current. As bursts continue, the effects of dynorphin accumulate, with a small increase per spike but a long half life (∼10 s), while [Ca^2+^]_i_ remains stable. Dynorphin weakens the ability of [Ca^2+^]_i_ to suppress the leak current, and, as a result, the burst becomes sensitive to small fluctuations in firing rate. A small drop in firing rate, within the range of random variation, can be sufficient to cause the plateau to collapse, causing the leak current to switch back on, silencing firing.

The silent period depends on the reverse order of effect between [Ca^2+^]_i_ and dynorphin. [Ca^2+^]_i_ decays more rapidly, so that the more prolonged effect of dynorphin is left unopposed; this causes a large leak current that hyperpolarises the cell and prevents burst initiation and most spike firing until dynorphin has sufficiently decayed. The cycle then repeats when enough spikes occur to initiate a new burst.

The key to the model parameters is to balance dynorphin's increase per spike and half life such that it does not quite reach equilibrium at the plateau firing rate. This leaves a gradual increase which eventually terminates the burst. More dynorphin release per spike or a slower decay will cause faster accumulation and terminate the burst more quickly. Too little per spike or too fast a decay and the effects of dynorphin will not continue to increase, so that the burst never terminates. Equally, [Ca^2+^]_i_ parameters must be such that [Ca^2+^]_i_ is sufficient to initiate and sustain a burst, but not so strong that it cannot be eventually overcome by dynorphin.

### Testing the model with antidromic spikes

**Figure 5:**
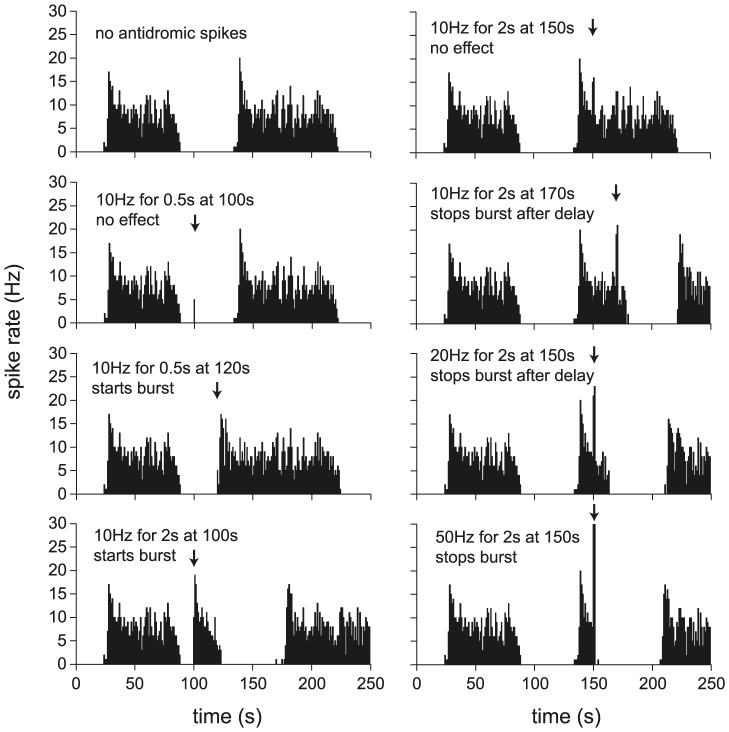
Using simulated antidromic spikes to trigger and terminate bursts. The data here uses the model fitted to cell v4, repeated using the same random synaptic input. Antidromic stimulation (as in [21]) is simulated by adding spikes to the model, at a specified frequency and time. In the left column, spikes are added during the silent period, attempting to trigger a burst. In the right column, spikes are added during the second burst, attempting to terminate the burst. Burst triggering is more likely when stimulated later into the silent period, or using a more intense stimulation. Generally, burst termination requires a more intense stimulation than burst triggering. Successful termination is more likely later into the burst, when there is more dynorphin accumulation, or with a more intense stimulation. The competing effects of spike-triggered increases in [Ca^2+^]_i_ and dynorphin cause a delay before termination occurs, unless the stimulation is sufficiently intense to trigger a large AHP, which immediately terminates spike firing.

*In vivo* experiments have shown that bursting can be both initiated and terminated by triggering increased spike firing, either by evoking spikes antidromically by electrical stimulation of the axons [4], or by stimulating increased synaptic input [20]. It is an important test of the model to be able to reproduce this, as these effects have clear implications for information coding. Figure 5 shows simulated antidromic spikes in a typical model cell. The model has the advantage that its noisy input activity can be repeated precisely, so that the effects of interventions can be tested against known times of burst initiation and termination. In the model cell illustrated, stimulating at 10 Hz for 0.5 s has no effect early in the silent period, but more intense stimulation (10 Hz for 2 s) or stimulation later in the silent period, causes early burst initiation. Triggering early burst termination requires stronger stimulation than burst initiation, but shows a similar pattern, requiring either a more intense stimulation (20 Hz for 2 s) or stimulation at a later point in the burst. There is still a delay before the burst stops, due to the opposing effects of [Ca^2+^]_i_ and dynorphin: the activity-induced increase in [Ca^2+^]_i_ sustains the burst before the longer lasting activity-dependent dynorphin increase causes termination. Such delayed terminations are an experimentally-observed feature of bursts that are truncated by modest stimulation (see Figure 3A of [20]). Applying a very intense stimulation (50 Hz for 2 s) causes a more rapid termination by increasing the AHP sufficiently to block spike firing.

### Model response to increased input

**Figure 6:**
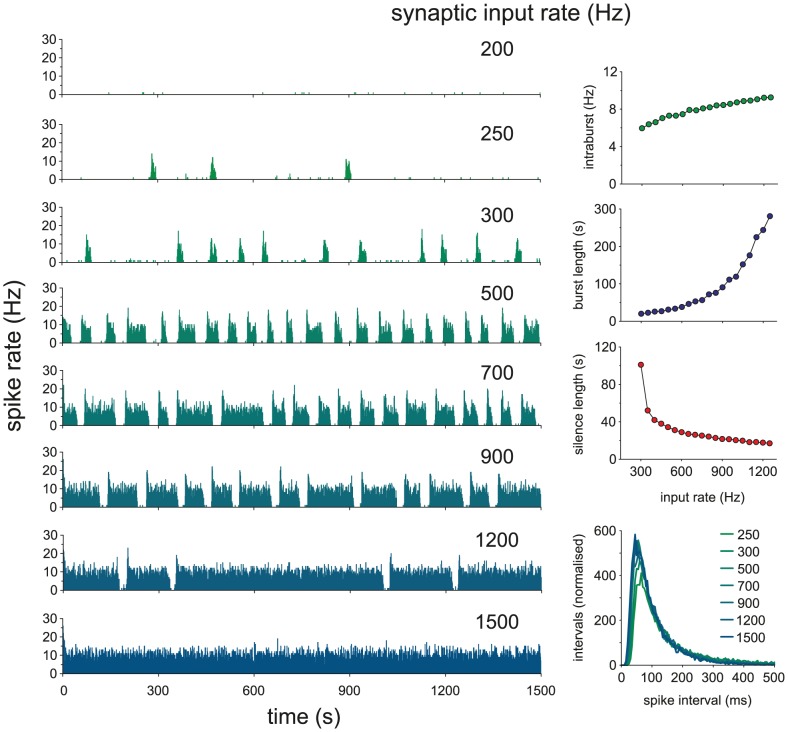
Model cell behaviour with increasing synaptic input. When osmotic pressure is increased *in vivo* we see a shift to phasic firing followed by increases in intraburst firing rate and burst duration, eventually shifting to continuous firing [3]. Here, we reproduce this in the model (using parameters fitted to cell v2) by increasing synaptic input. Intraburst firing rate increases fairly linearly, whereas the increase in burst duration is much more non-linear. Silence duration shows a fairly linear decline after phasic firing is established.

*In vivo*, when osmotic input is increased, an increased proportion of vasopressin neurons fire phasically, shifting from slow irregular firing. Phasic neurons also show longer bursts, shorter silences, and higher intra-burst firing rates [3], eventually shifting to continuous firing. We tested this in the model by increasing the synaptic input rate. Similarly, model cells progress from slow sparse firing to short irregular bursts and then full phasic firing with bursts increasing in duration and firing rate and shortening silent periods, until they eventually shift to continuous firing (Figure 6). The increase in intraburst firing rate is more linear than the increase in burst duration due to the additional opposing effect of the AHP on firing rate.

### Simulating the effect of hypertonic injection

The experimental study of Brimble and Dyball [26] reports the responses of vasopressin cells to a systemic injection of hypertonic saline, which triggers a rapid and prolonged increase in osmotic pressure. In that study, relatively slow phasic neurons shifted to longer, faster bursts, and faster firing phasic neurons shifted to continuous firing, similar to our results above. However, non-phasic slow, irregular neurons when challenged go through a transitional period of ∼10 min of continuous firing before shifting to stable phasic firing (see Figure 5 in [26]). The firing rate of the neurons rises much more quickly than the shift to bursting, suggesting that the change in firing pattern is determined by the state of the burst mechanism rather than the input *per se*.

**Figure 7:**
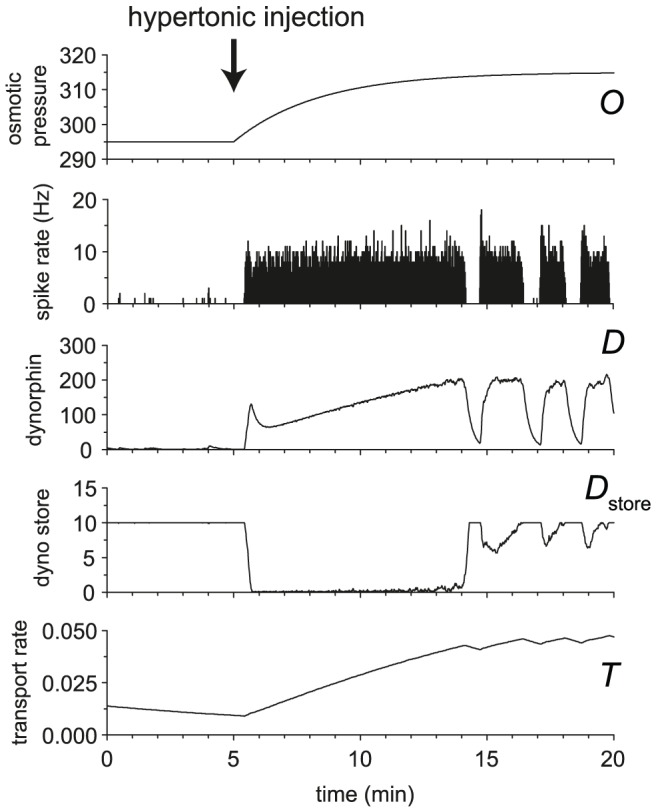
Using the model to simulate the effect of hypertonic saline injection. An injection of hypertonic saline causes a rapid increase in osmotic pressure. The rapid increase in input causes some initially slow firing vasopressin cells to shift immediately to fast continuous firing before settling in a phasic pattern after a long delay (∼10 min) [26]. We hypothesise that this is due to insufficient availability of dendritic dynorphin, which takes time to upregulate. We tested this using an extension to the basic model and were able to reproduce the effect observed *in vivo*. Osmotic pressure was initially set at 295 and increased to 315 by injection at 5 min. We suggest that releasable dynorphin store upregulation is dependent on a slow activity-dependent vesicle transport mechanism, *T*. The extended dynorphin mechanism uses parameters *k_T_* = 0.00001, λ*_T_* = 500000, *k_D_*_store_ = 0.02, λ*_D_*_store_ = 1000000, *D*_spike_ = 0.1, *D*_storecap_ = 10, and τ*_O_* = 200000.

We simulated the delayed effect of hypertonic saline injection on the rise in osmotic pressure, but could not reproduce these experimental results with our basic model. However, the pool of readily-releasable vesicles in the dendrites of magnocellular neurones is labile, and regulated in an activity-dependent manner. We therefore hypothesised that, in the absence of activity-dependent replenishment of the readily releasable pool, the initial lack of burst termination might be due to insufficient readily releasable stores of dynorphin. Out first attempt at modelling this used a simple mechanism where spike-triggered dynorphin release was directly dependent on a dynorphin store charged by spike activity. However this resulted in shorter, not longer, bursts as input activity increased, and so we developed a more complex mechanism where spike triggered dynorphin release is partially decoupled from the dynamics of the releasable dynorphin store, and using this mechanism we were able to reproduce the initial period of continuous firing before onset of phasic firing (Figure 7). In this mechanism, a slowly accumulating measure of spike activity (*T*, hypothesised to represent slow activity-driven vesicle translocation) determines the rate at which the readily releasable pool of dynorphin is replenished. While *T* is too low, the release decoupling element (*r* in eqn. 12 and 13) makes some spikes fail to trigger dynorphin release, slowing the increase in dynorphin while store replenishment is too slow to keep up with the spike rate. This results in a gradual increase in the amount of dynorphin available for activity-dependent release until it reaches an equilibrium with activity-dependent depletion, at which point it is sufficient to sustain phasic firing.

### Simulating the effect of the dynorphin antagonist nor-BNI

**Figure 8:**
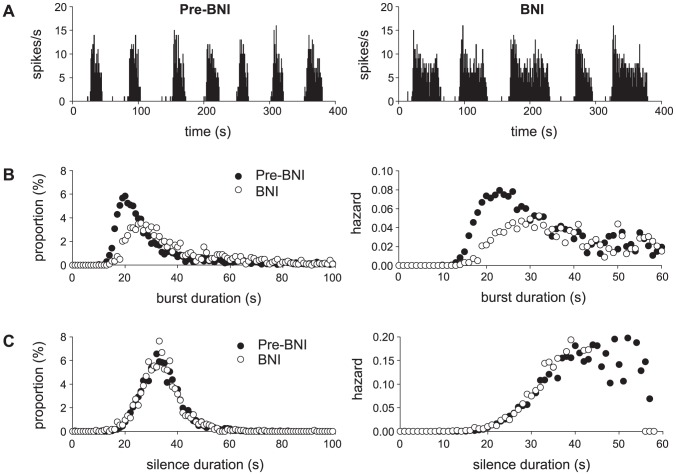
Simulating *in vivo* study of the effect of reduced dynorphin on burst and silence duration. Our data simulates in vivo analysis using data from multiple cells by generating 100 model cells with random variation in the seven non-synaptic parameters used to fit the varied cells in Figure 3 (Table 4). The dynorphin antagonist was simulated by reducing k*_D_* by 15% in each generated cell. This is sufficient to have a large effect on spike rate (A) and the burst duration histogram and hazard (B), comparable to the *in vivo* results in Brown et al 2006 [28] (c.f. their figure 5) but also shows very little effect on the silence (inter-burst interval) duration (C), similar to what they observe, suggesting that their results do not, as they interpret, exclude a role for dynorphin in generating inter-burst silence.

In the model, one of the main elements which generates the inter-burst silence is a dynorphin driven hyperpolarisation. In addition to dynorphin, silence duration is also determined by the AHP and the synaptic input rate. However, Brown et al [28] studied the effect of the competitive dynorphin antagonist nor-BNI, combining analysis of *in vitro* and *in vivo* results to suggest that dynorphin does not affect inter-burst activity. We used the model to test this by simulating their *in vivo* results. As a competitive antagonist, nor-BNI reduces rather than blocks dynorphin's effect and we simulated this in the model by reducing parameter k*_D_* (eqn. 7) sufficiently to produce a similar increase in burst duration as they observe. Their *in vivo* data is taken from multiple cells, and to simulate cell variation we generated 100 model cells based on the fit to cell v1, with small random variations in the parameter subset we varied to fit different *in vivo* cells (Table 4). We ran each cell for 3000 s with and without reduced k*_D_*, generating a total of 3032 bursts under simulated control, and 1239 bursts under simulated nor-BNI. We then generated the same collected data burst and silence duration histograms and hazards as shown in Figure 5 of Brown et al 2006 [28] (Figure 8).

**Table 4:** Parameters varied to simulate *in vivo* cell heterogeneity.

Parameter	*I*_re_	λ*_HAP_*	*k_DAP_*	*k_AHP_*	*k_C_*	*k_D_*	λ*_D_*	*g_L_*
mean	600	9	0.50	0.00012	11	2.7	7500	8.5
S.D.	0	1	0.25	0.00004	1	0.3	0	1.0

Our results show a similar strong effect of reduced dynorphin action increasing burst duration, but despite our dynorphin-based mechanism, we also show very little effect on silence duration, similar to the result of Brown et al [28]. Further reductions in dynorphin effect (not shown) do show a reduction in the mean silence duration, but it is much smaller than the effect on increased burst duration, and in a varied population results in many cells shifting to continuous firing.

### Phasic firing and population signal encoding

We want to understand what use asynchronous phasic firing is, in particular what advantage it gives to information processing in a neuronal population. We can use the model to study this, running duplicates of the single neuron model in parallel, and taking the summed spike activity as the population output.

**Figure 9:**
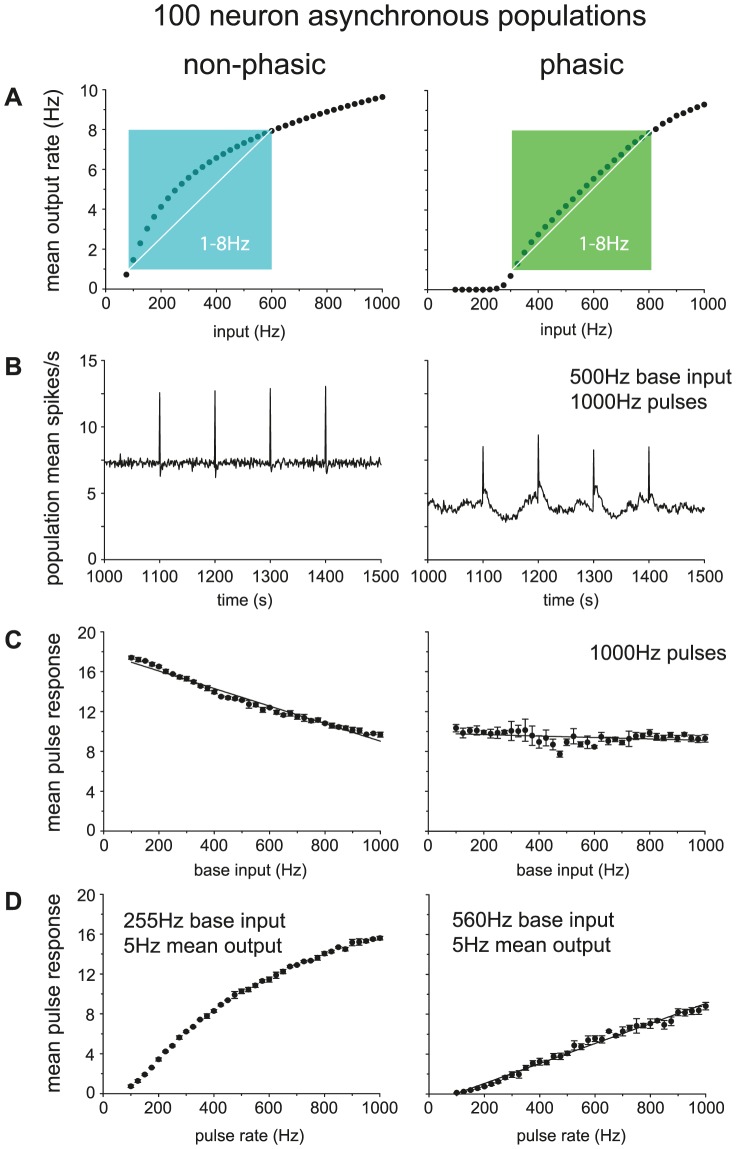
Comparing the population spike response of phasic and non-phasic model cells. Data here is from a population of 100 identical model cells (using the fit to cell v1) run in parallel, taking as output the summed spike rate. Synaptic input uses a common rate parameter, *I*_re_, but was generated independently for each neuron, maintaining an asynchronous population. Non-phasic cells use g*_L_* = 0. To test pulse response, *I*_re_, was set to a defined pulse input for 1s at four time points spaced 100s apart. The pulse response was measured as the mean population spike rate response over the four pulses. (**A**) Mean population rate with a varied base input rate (range 100–1000). The population response in the phasic cells, shows a much more linear relation to the input, once input is sufficient to trigger phasic firing, particularly in the physiological spiking range of 1–8Hz. (**B**) Population spike activity in non-phasic and phasic cells with matched 500Hz base input rate and 1000Hz pulse input. The summed asynchronous bursting activity in the phasic cells does not show the bursting, but does show more variable activity and a lower mean spike rate. Each input pulse produces a distinct increase in firing rate. (**C**) Testing response with a varied base input rate (range 100–1000) and fixed 1000Hz pulse input. As background input increases, the mean pulse response in the non-phasic cells gradually falls, whereas in the non-phasic cells it is much more consistent. (**D**) Testing response with varied pulse input rate (range 100–1000Hz) and base rate set to give matched mean firing rate = 5 spikes/s (non-phasic base *I*_re_ = 255, phasic base *I*_re_ = 560). The phasic population response shows a much more linear relation to the input pulse rate.

To study population response, we compare the summed activity of 100 model cells in two conditions: one where all cells were made phasic by setting *g_L_* = 8.5, and one where the cells were made non-phasic by setting *g_L_* = 0, but with no other change (Figure 9). To maintain an asynchronous population, we use independently generated input PSPs for each model cell, using a common input rate, parameter *I*_re_.

We first measured the mean spike rate in the population in response to constant input rates over the range 100–1000 Hz (beyond this range the phasic cells begin to fire continuously and behave as non-phasic cells), running the model cells for 2000 s of activity (Figure 9A). The non-phasic cells show a very non-linear relationship between input and output rates, with an initially steep relationship at low input rates that flattens at higher input rates. By contrast, the phasic cells show very little response until the input is sufficient to trigger bursting, but then show a much more linear average increase in response. In the overall output range of ∼1–8 spikes/s (including mean intraburst rates as high as 15spikes/s), which corresponds to the normal physiological dynamic range of firing activity of vasopressin cells *in vivo*, the relationship is particularly linear for phasic cells compared to non-phasic cells. At high input rates, the phasic population shifts to continuous firing, at which point they give a non-linear response that is similar to that of the non-phasic cells. Thus overall, the phasic cells show a strong linearization of the response to increasing input, compared to the non-phasic cells, which show a typical non-linear neuronal response.

To study how the population responds to transient pulses, we challenged the two populations with four 1-s duration periods of increased input by setting parameter *I*_re_ to 1000 Hz at 100-s intervals, testing over the same range of background input as Figure 9A. The phasic population shows more variability in its average output activity, but the response to the input pulses is similar to that of the non-phasic population (Figure 9B). However, unlike the non-phasic population, the phasic population responds to pulses in a way that is relatively independent of the background input, producing a consistent response that is largely independent of background rate (Figure 9C). By contrast, for the non-phasic population, the mean response to pulses reduces as the background input increases, as the constant firing increases the amount of time that cells are refractory due to activity-dependent hyperpolarisation.

Finally, we tested the response of the two populations to transient input of varying amplitude. For this, we fixed the background input (255 Hz for non-phasic, 560 Hz for phasic) so that the two populations were firing at the same mean rate (5 Hz) and tested the effects of input pulses in the range 100–1000 Hz. The phasic population (Figure 9D) responds to increasing pulse amplitudes with a linear average increase in firing rate (measured during the pulses). By contrast, the non-phasic population again shows a non-linear response. The response of the phasic population to transient pulses is also smaller than that of non-phasic cells, supporting the previous suggestion [20] that the asynchronous phasic cells function as a low-pass filter.

## Discussion

We have described an integrate-and-fire based model of magnocellular vasopressin neurons, which is capable of explaining and reproducing a large range of the firing behaviours observed *in vivo*. The model implements a phasic firing mechanism based on a hyperpolarising K^+^ leak current, modulated by intracellular calcium and dynorphin.

The most important advance with this model is that it can realistically reproduce the dynamic behaviour of vasopressin cells as synaptic input changes, giving the ability to go forward and test how the neurons' properties relate to their function. Here, we have begun to use the model to test the general information processing properties of phasic firing neurons. In particular, we asked what are the specific consequences of phasic firing for information coding, given that phasic vasopressin cells fire asynchronously. We approached this question by constructing two populations of neurons that matched the properties of vasopressin cells closely, and which differed only in their ability to fire phasically. We showed that, compared with non-phasic cells, phasic cells respond (as a population) to sustained increases in synaptic input with a relatively linear increase in mean firing rate in the range 0–10 spikes/s. This is the physiological dynamic range of vasopressin cells: 10 spikes/s is about as fast as these cells can sustain firing under extremes of physiological dehydration [2]–[4].

Previously we suggested that a population of asynchronous phasic neurons might function as a low-pass filter [20], as short pulses of increased input will sometimes start and sometimes stop bursts. Testing with the model shows that non-phasic neurons are indeed much less sensitive to transient increases in synaptic input (Figure 9D), although with sufficiently large transients there is still some increase in activity, as the increase in spikes from starting a burst is greater than the reduction in spikes from stopping one. The net effect also includes cells where the input pulse generates extra spikes without stopping a burst. Moreover, whereas the response of non-phasic cells to brief transients strongly depends on the background level of synaptic input, phasic cells respond to transient increases in a way that is largely independent of background synaptic input. Finally, the response of phasic cells to transient perturbations is again much more linear with the magnitude of those perturbations, than that of non-phasic cells. This idea, that competing excitatory and inhibitory effects lead to overall linearization of the response is comparable to our previous result [19] showing that mixed excitatory and inhibitory synaptic input gives a more linear firing response in neurons.

Individually, phasic cells are extremely non-linear and inconsistent in their responses to changes in afferent input [2], however, here we have shown that a population of asynchronous phasic cells is much more linear and more consistent in its overall response than a matched population of cells that do not display phasic firing. The natural inference is that these consequences of phasic firing are adaptive, and constitute an explanation of why vasopressin cells fire phasically.

### The model

The present model is relatively simple; it has 21 parameters, but five of these define the synaptic input, and only one of these, *I*_re_, is varied here, to control the input rate. Parameter *C*_rest_ is only included to scale [Ca^2+^]_i_ to *in vivo* units, and can be removed by setting to 0. Several others can be fixed, and only eight varied parameters are required to fit the model to a wide range of recorded cells.

The one element of the model which was predicted, rather than directly based on experimental data, is the fast DAP, which is required to fit the short term spike patterning detected in the ISI histogram and hazard function. It turns out that such a current has already been found in recent *in vitro* work of Armstrong *et al* [29]. Our predicted 150 ms half life is a close match to their measured value of 200 ms.

Both a medium-duration (∼500 ms half life) and a slow AHP have been found in vasopressin cells [27], [30]. We represent the AHP in the model using only a single slow AHP. We tested whether adding a second AHP would alter the model behaviour (not shown) but found no clear effects of this. The very long half life (10s.) is necessary to fit the duration of the peak at the head of each burst. The Roper model uses a single medium-duration AHP but also shows burst peaks which are much shorter than *in vivo*.

One of the distinctions of the model from previous published attempts is that it works with very simple dynorphin dynamics. The Nadeau spiking model was unable to reproduce the increase in burst duration with increased input activity, and they corrected this by adding frequency-dependent fatigue to the dynorphin signal. The Roper model itself added spike rate based facilitation to dynorphin accumulation, attempting to make burst duration less regular. Our model is able to produce the increased burst duration with a simple fixed rate of dynorphin accumulation per spike, suggesting that the more complex dynamics are not required. It will require further work, attempting to map between the models, to understand what the important difference is.

We did use more complex dynorphin dynamics when attempting to simulate the effect of sudden changes in osmotic input, showing that the observed switch to continuous firing from slow irregular firing, before settling into phasic firing, can be explained by activity-dependent upregulation of releasable dendritic dynorphin stores. In vasopressin neurons, dynorphin is co-packaged with vasopressin in large dense-cored vesicles, and these vesicles can be released from the dendrites by calcium-dependent exocytosis. Electrical activity can induce dendritic release through voltage-gated calcium entry through mainly N-type calcium channels [31], but the amount of release in response to electrical activity depends on other factors. In particular, dendrites possess a readily-releasable pool of vesicles close to the plasma membrane [32], and recruitment of vesicles into this pool from deeper reserve stores is regulated by the cytosolic actin cytoskeleton in a calcium-dependent way [33], allowing for a slow activity-dependent augmentation of dendritic release. Adding this mechanism only affects model behaviour in response to sudden and prolonged changes to input activity. We don't require the more complex mechanism to simulate other behaviour and so consider this an optional extension for the purposes of further work. It is an advantage to maintain a model which is as simple as possible in order to understand its behaviour.

The obvious simplification in our model, compared to the Hodgkin-Huxley based models, is that we don't have voltage dependency. During the model's development, voltage dependency was tested with the K^+^ leak current, but was unable to produce proper burst activity. Experience suggests that an incomplete implementation of voltage dependence does not work well in neural models. The HAP for example, modelled as a decaying exponential, requires a very large initial magnitude (60 mV) when the spike is just a point event. In an integrate-and-fire model this doesn't matter – the HAP is unrealistically large initially, but only at a time when cells are refractory, and so has no effect on spike patterning. However, if other voltage-dependent elements are added then the large voltage perturbation can cause unrealistic behaviour. We would argue that it is more important for an *in vivo* model to match spike patterning, rather than the detailed dynamics of individual spikes – as generally the experimental data *in vivo* essentially capture only spike events. Obviously spike events and membrane voltage changes are related, but the detailed parameters are more difficult to measure *in vivo*, and voltage dependence *in vivo* is much weaker than *in vitro* [17]. The deafferentation of neurons that is inevitable in the preparation of hypothalamic slices for *in vitro* recordings leaves the cells relatively denuded of synaptic input; accordingly the cell input resistance is inevitably higher due to the lack of activation of neurotransmitter-gated ion channels, and the higher input resistance amplifies the effects of conductance changes on membrane potential. Comparing *in vivo* and *in vitro* recordings of vasopressin cells shows a large difference in post-spike excitability [18], that cannot be accounted for by the membrane voltage effects of a different level of background synaptic input, indicating that channel dynamics are very different *in vivo*. The combination of losing the stochastic element of the synaptic input and the larger post-spike potentials makes *in vitro* spiking slower and more regular, as observed in the ISI histograms [18]. In addition, the slower, larger DAP makes bursting regenerative. Bursts become self-sustaining, and not subject to external input. *In vivo*, bursts are generated by the same intrinsic mechanism, but the smaller DAP is not sufficient on its own, requiring synapse driven depolarisation to maintain a burst. Thus *in vivo* bursting characteristics depend on both synaptic input and the intrinsic bursting mechanism.

### Cell heterogeneity and fitting the model

Vasopressin neurons display diverse patterns of spontaneous spike activity; some cells are relatively silent or irregular, some are continuous, and the rest show variations of the phasic pattern, with varying intraburst rates, burst durations and silence durations. We have shown (Figure 6) that these different modes of spiking can be produced in a single model neuron by varying the synaptic input rate. However, other parameter changes were required to reproduce the different phasic firing characteristics observed between cells. To determine the subset of parameters essential for capturing the full heterogeneity seen *in vivo*, we attempted to fit different cells changing as few parameters as possible (Table 2). In addition to the synaptic input rate (*I*_re_), the HAP and fast DAP parameters were required to fit the short-term spike patterning reflected in the ISI histogram and hazard function. The most important parameters however were *k_AHP_*, and the K^+^ leak parameters, in particular *k_D_*, which determines dynorphin's effect on the slow DAP. The AHP is essential to determining the intraburst spike rate, balancing against the depolarising effects of the DAPs. Dynorphin's most sensitive effect is on limiting burst duration, which in turn defines how much input is required to shift a cell to continuous firing, but in the model it is also essential to determining mean silence duration, as further discussed below. To fit the cells with longer mean silence, we had to use a longer dynorphin half-life value, λ*_D_*.

### Comparison with other work

Nadeau et al [34] have recently extended their spiking model to include the vasopressin secretion response, and have used their model to explore what properties of the spiking mechanism might underlie the heterogeneity observed in the cell population. The core of their secretion model is based on an interpolation of *in vitro* data which demonstrated the frequency facilitation effect [9], assuming that this is sufficient to also represent the fatigue effect on secretion. The important test of a secretion model should be whether it can reproduce the enhanced secretion response to phasic firing compared to continuous firing at the same mean spike rate [6]. This is thought to depend on both the facilitation and the fatigue elements of the secretion mechanism.

Their spiking model, like our present model, includes a dynorphin mechanism that plays a role in cell heterogeneity, and which is a key element in determining whether cells fire irregularly, phasically or continuously. They suggest however that synaptic input in the normal frequency range is not a factor in determining firing mode, and their model shows very little response to increases in synaptic activity while in the phasic firing mode. The differences between the Nadeau model and the present model mirror the differences between vasopressin cell activity *in vitro* and *in vivo*: the Nadeau model is a regenerative spiking model, in which a relatively very low level of synaptic activity provides a limited variability in spike timing, while the present model displays spike activity that is wholly dependent on a relatively high level of synaptic input. In response to increased osmotic pressure, their individual cells produce a step-like increase in spike rate and secretion. They suggest that the linear population response is based on inter-cell variation in the dynorphin parameters and resting potential, so that the proportion of active cells gradually increases with increased osmotic input. Such a non-linear increase is not observed in our model, nor in the responses of individual vasopressin cells to progressive osmotic pressure changes *in vivo*.

We further tested our model by using it to simulate various *in vivo* experiments which have studied the spiking activity and the underlying mechanisms. We predict that some slow activity driven dendritic vesicle translocation is responsible for the delayed shift to phasic firing in response to a sudden rise in osmotic input. We do not require this mechanism for other results, and so retain it as an optional extension to the model, but it may play an important role when further considering the effects of dendritic release in future work, and has parallels to the mechanisms postulated for the axonal secretion, the idea of a releasable and a reserve store [25].

It is now widely accepted that dynorphin is an essential element of the phasic mechanism, and determines burst duration, but there is debate on what role it plays in determining inter-burst silence. Combining *in vitro* and *in vivo* analysis of the response to a dynorphin antagonist, Brown et al [28] suggested that dynorphin does not play a role. However, our model, in which dynorphin is a part of the silence generating mechanism, is able to replicate the results of their *in vivo* analysis. We show that reducing the effect of dynorphin has a much larger effect on burst duration than silence duration. It does reduce silence duration, but to show this significantly requires a dose of the antagonist which will turn most cells continuous. The cells selected for their analysis were by nature those which retained bursting, and likely to show less effect on silence duration. Their in vitro data shows no change in time course of the inter-burst hyperpolarisation with reduced dynorphin. We would suggest that this is due to the slow AHP acting on a similar time course. More recent *in vivo* results [35] do show a reduction in silence duration in response to the same dynorphin antagonist, supporting the hypothesis that dynorphin drives a post-burst hyperpolarisation.
